# NMDA Receptor Antagonist Memantine Ameliorates Experimental Autoimmune Encephalomyelitis in Aged Rats

**DOI:** 10.3390/biomedicines12040717

**Published:** 2024-03-23

**Authors:** Biljana Bufan, Ivana Ćuruvija, Veljko Blagojević, Jelica Grujić-Milanović, Ivana Prijić, Tatjana Radosavljević, Janko Samardžić, Milica Radosavljevic, Radmila Janković, Jasmina Djuretić

**Affiliations:** 1 Department of Microbiology and Immunology, Faculty of Pharmacy, University of Belgrade, 11000 Belgrade, Serbia; biljana.bufan@pharmacy.bg.ac.rs; 2 Department of Research and Development, Institute of Virology, Vaccines and Sera, Torlak, 11000 Belgrade, Serbia; ivanajakovljev1@gmail.com (I.Ć.); veljko.blagojevic1988@gmail.com (V.B.); ivanavujnovic@hotmail.com (I.P.); 3 Institute for Medical Research, National Institute of the Republic of Serbia, Department of Cardiovascular Research, University of Belgrade, 11000 Belgrade, Serbia; jeca@imi.bg.ac.rs; 4 Institute of Pathological Physiology, Faculty of Medicine, University of Belgrade, 11000 Belgrade, Serbia; tatjana.radosavljevic@med.bg.ac.rs; 5 Institute of Pharmacology, Clinical Pharmacology and Toxicology, Faculty of Medicine, University of Belgrade, 11000 Belgrade, Serbia; jankomedico@yahoo.es (J.S.); milica.radosavljevic.bg@gmail.com (M.R.); 6 Institute of Pathology, Faculty of Medicine, University of Belgrade, 11000 Belgrade, Serbia; radmila.jankovic@med.bg.ac.rs; 7 Department of Pathobiology, Faculty of Pharmacy, University of Belgrade, 11000 Belgrade, Serbia

**Keywords:** aging, NMDARs, memantine, EAE, multiple sclerosis

## Abstract

Aging is closely related to the main aspects of multiple sclerosis (MS). The average age of the MS population is increasing and the number of elderly MS patients is expected to increase. In addition to neurons, *N*-methyl-D-aspartate receptors (NMDARs) are also expressed on non-neuronal cells, such as immune cells. The aim of this study was to investigate the role of NMDARs in experimental autoimmune encephalomyelitis (EAE) in young and aged rats. Memantine, a non-competitive NMDAR antagonist, was administered to young and aged *Dark Agouti* rats from day 7 after immunization. Antagonizing NMDARs had a more favourable effect on clinical disease, reactivation, and apoptosis of CD4^+^ T cells in the target organ of aged EAE rats. The expression of the fractalkine receptor CX3CR1 was increased in memantine-treated rats, but to a greater extent in aged rats. Additionally, memantine increased Nrf2 and Nrf2-regulated enzymes’ mRNA expression in brain tissue. The concentrations of superoxide anion radicals, malondialdehyde, and advanced oxidation protein products in brain tissue were consistent with previous results. Overall, our results suggest that NMDARs play a more important role in the pathogenesis of EAE in aged than in young rats.

## 1. Introduction

Aging exerts a great impact on the main aspects of multiple sclerosis (MS): the clinical course, the pathological and immunological processes, and the therapeutic choices [1]. The most common age for onset of MS is between 20 and 40 years. In young patients, the predominant MS phenotype is relapsing–remitting disease. However, primary progressive MS tends to occur later (mean age 45 years) [2,3]. In recent years, numerous data suggest that: (1) the proportion of patients with late-onset (50 years or older) and very late-onset (60 years or older) MS has increased significantly, despite a shortened time to diagnosis, (2) the mean age at diagnosis has shifted toward older age, (3) the survival rate of all MS patients (both early and late onset) has increased, and (4) the age at onset of disease progression is similar (around 45 years of age) for primary and secondary progressive forms of MS, i.e., the onset of progressive disease course appears to be age- rather than disease-duration-dependent [4,5,6,7,8,9]. Life expectancy is only modestly affected with MS [10]. The increased prevalence of MS is largely due to the increased life expectancy of the elderly and thus more disabled MS population. Aging of both the immune and central nervous systems may contribute to the progression of MS and could be associated with poor response to current disease-modifying treatments (DMTs) or increased risk of side-effects [2]. Patients over 50–60 years of age are underrepresented in clinical trials, making it very difficult to determine whether available treatments are safe and effective for older people. Several meta-analyses suggest that DMTs have no benefit for disability progression over age 53, and that the risk of neoplasms with depletive agents increases over age 45 [4,11,12]. Age-related incidence and clustering of comorbidities may affect the efficacy and safety of DMTs [4]. It is therefore extremely important to investigate aging-related mechanisms involved in pathogenesis and progression of MS.

Aging affects N-methyl-D-aspartate receptors (NMDARs), their expression and function, expression of their subunits, and their composition within various brain regions. Moreover, NMDARs are more susceptible to aging than other glutamate receptors. The NMDAR is a heterotetrameric ionotropic glutamate receptor consisting of combinations of the GluN1, GluN2, and GluN3 subunits [13,14]. It is assumed that the GluN2B subunit is most affected by the aging process, and shows a dramatic decrease in mRNA expression [13]. In addition, the amyloid precursor protein influences the expression of GluN2B in synapses. Changes in the processing of the amyloid precursor protein with aging may lead to synaptic dysfunction and an increased risk of neurodegeneration [15].

As well as on neuronal cells, NMDARs are also found on non-neuronal cells such as endothelial, microglial, and immune cells [16,17,18]. Stimulation of NMDARs in lymphocytes affects the performance of these cells since it has been shown that the application of NMDAR antagonists is able to inhibit T cell proliferation by preventing their activation [19]. It has been demonstrated that, in humans, NMDARs are rapidly upregulated after CD4^+^ T cell activation. Moreover, the functions of Th1 and Th2 cells, such as proliferation, cytokine production, and cell survival, are differentially affected by NMDAR agonists [20]. Experiments in vivo found that, during development of inducible inflammation of the spinal cord (SC) of rats, the inflammatory area is infiltrated only by those lymphocytes that express NMDARs [21].

It has been shown that the glutamate level in the CNS of MS patients increases [22]. Over-activated NMDARs due to elevated glutamate levels trigger substantial calcium influx, leading to calcium overload in both cells and mitochondria. Calcium overload mediated by NMDARs causes mitochondrial dysfunction and oxidative stress, initiating pro-inflammatory pathways [22,23]. Consistent with the previous, antagonists of the glutamatergic NMDARs, such as memantine, reduce the severity and duration of neurological deficits in experimental autoimmune encephalomyelitis (EAE), a well-established animal model of multiple sclerosis [24].

The aim of the present study was to investigate the impact of aging on the role of NMDARs in the pathogenesis of EAE using memantine, a non-competitive NMDAR antagonist that limits pathological activity of NMDARs while preserving normal synaptic activity [25]. The animal model of MS, EAE, was induced in *Dark Agouti* (*DA*) rats. Young adult *DA* rats show high susceptibility to EAE, while aging reduces the incidence of EAE [26,27], which is similar to the effects of aging on the incidence of MS. Our results reveal an age-dependent effect of NMDARs on neurological scores of EAE, on SC infiltration by CD4^+^ T lymphocytes and their reactivation, CX3CR1 expression by microglia, nitric oxide (NO) production by macrophages/microglia, and free radical generation and antioxidant protection in rat brain tissue.

## 2. Materials and Methods

### 2.1. Experimental Animals and EAE Induction

Young adult (2–3-month-old) and aged (22–24-month-old) female *DA* rats were used in this study. All rats were from breeding colonies at the Department of Research and Development, Institute of Virology, Vaccines and Sera in Belgrade. The animals were kept in accordance with standard laboratory conditions, with their health monitored daily. None of the animals involved in the experiments exhibited any clinical signs of neural disorders, and, upon necropsy, none of the aged rats displayed any visible signs of illness. All procedures conducted on the animals and their care adhered strictly to the guidelines outlined in Directive 2010/63/EU of the European Parliament and of the Council on the protection of animals used for scientific purposes. Animal manipulation and experimental procedures were approved by the local Ethics Committee (Ethical Committee of the University of Belgrade-Faculty of Pharmacy; permit no. 1667/2). All experiments followed ARRIVE 2.0 guidelines for reporting animal research [28]. EAE was induced with rat SC homogenate (SCH) in phosphate-buffered saline (PBS, 50% *w*/*v*) mixed with an equal volume of complete Freund’s adjuvant (CFA) containing 1 mg/mL of heat-killed and dried *Mycobacterium tuberculosis H37Ra* (Sigma-Aldrich Chemie GmbH, Taufkirchen, Germany). Each rat was injected intradermally in the hind footpad with 100 µL of the emulsion SCH+CFA, followed by a subcutaneous injection of 0.25 mL of 5 × 10^8^ *Bordetella pertussis* (Institute of Virology, Vaccines and Sera “Torlak”, Belgrade, Serbia). In *DA* rats, this immunization protocol results in a single-phase disease followed by complete recovery. The severity of neurological impairment and changes in body weight were assessed daily by two experienced observers who were blinded to the treatment administered to the animals. Neurological impairment was graded as follows: 0 for no clinical signs; 0.5 for distal tail atony; 1 for flaccid tail; 2 for hind limb paresis; 3 for complete hind limb paralysis, and 4 for complete paralysis of all limbs or a moribund state. All animals showing signs of disease were provided with easy access to mashed food and water.

### 2.2. Experimental Design

Rats of both ages were randomly assigned to either memantine treatment or vehicle (sterile PBS) administration. Memantine (M9292, Sigma-Aldrich Chemie GmbH, Taufkirchen, Germany), a non-competitive NMDAR antagonist, was administered at a dose of 60 mg/kg body weight (bwt)/day for 7 consecutive days (7th–13th day post-immunization) by oral gavage to young and aged EAE rats. Administration of the drug at 60 mg/kg bwt/day was demonstrated as the optimum dose, significantly reducing disease and lesions in EAE [29]. Control EAE rats were administered an equivalent volume of vehicle daily by oral gavage. At the peak of the disease 13th day post-immunization (dpi), animals were anesthetized and perfused with PBS. Six rats per group per experiment were used. Following the perfusion, mononuclear cells (lymphocytes, monocytes, microglia, and dendritic cells) were isolated from the SC and analyzed by flow cytometry (FCA) or stimulated overnight with 1 µg/mL of lipopolysaccharide (LPS; Sigma-Aldrich Chemie GmbH). Flow cytometry was used for assessment of lymphocyte apoptosis using the FITC Annexin V Apoptosis Detection Kit. Brain tissue was immediately frozen in liquid nitrogen and stored at −70 °C for later analysis. Redox status parameters were measured in brain homogenates. Evaluation of mRNA expression levels of transcriptional factor nuclear factor (erythroid-derived 2)-like 2 (Nrf2), heme oxygenase 1 (HO-1), and superoxide dismutase (SOD) 1 and SOD2 in brain tissue was performed by qRT-PCR.

### 2.3. Histopathological Analysis

After perfusion, the spinal cords (SCs) were extracted and divided into three segments (cervical, thoracic, and lumbosacral), and then immersed in 4% paraformaldehyde overnight for fixation. Transverse 5 μm thick sections of the paraffin-embedded tissue were stained with hematoxylin and eosin and assessed for signs of inflammation using the following scoring criteria: 0 for no inflammatory cells, 1 for a few scattered inflammatory cells, 2 for the formation of inflammatory infiltrates around blood vessels, and 3 for extensive perivascular cuffs with extension into adjacent subarachnoid space and SC parenchyma [26]. Every 50th transverse section of the spinal cord was examined for signs of inflammation. The sections were captured on an Olympus BH2 microscope equipped with camera.

### 2.4. Isolation of Spinal Cord Mononuclear Cells

To isolate mononuclear cells, SCs were homogenized using a 70 μm nylon cell strainer (BD Biosciences, Erembodegem, Belgium) and suspended in ice-cold RPMI 1640 medium (Sigma-Aldrich Chemie GmbH) supplemented with 5% fetal calf serum. The SC cell suspension was centrifuged using 40%/70% gradient of Percoll (Sigma-Aldrich Chemie GmbH). After centrifugation, the mononuclear cells were collected from the interface. Cell counts were determined using a Neubauer hemocytometer and 0.2% trypan blue solution to identify viable cells.

### 2.5. Cell Staining and FCA

#### 2.5.1. Antibodies and Immunoconjugates

For immunolabeling, the following monoclonal antibodies (mAbs) were used: phycoerythrin (PE)-conjugated anti-CD4 (clone OX-38), biotin-conjugated anti-CD45 (clone OX-1), biotin-conjugated anti-CD134 (clone OX-40), peridinin-chlorophyll-protein (PerCP)-conjugated anti-TCRαβ (clone R73), FITC-conjugated anti-IFN-γ (clone DB-1), and PE-conjugated anti-IL-17A (clone TC11-18H10). These mAbs, as well as PerCP-conjugated streptavidin, APC-conjugated streptavidin, and PE-conjugated donkey anti-rabbit F(ab)2, as second-step reagents, were obtained from BD Biosciences Pharmingen (Mountain View, CA, USA). FITC-conjugated anti-CD11b (clone ED8) was purchased from Serotec (Oxford, UK), APC-conjugated anti-CD4 (clone OX-38) from eBioscience (San Diego, CA, USA), and rabbit anti-CX3CR1 polyclonal Ab from Abcam (Cambridge, UK).

#### 2.5.2. Cell-Surface and Intracellular Antigen Immunostaining

Cells were incubated with saturating concentrations of fluorochrome-conjugated mAbs for 30 min at 4 °C in the dark, washed, and collected for FCA. In the case of biotin-conjugated/unconjugated Abs, the cells were incubated for a further 30 min with appropriate reagents for the second step. To determine cell apoptosis, after labeling of the surface markers, SC cells were washed with PBS and then with Annexin V binding buffer (BD Biosciences Pharmingen). After washing, the cells were incubated with 5 μL of Annexin V-FITC (BD Biosciences Pharmingen) for 15 min at room temperature in the dark and collected for FCA. For IL-17 and IFN-γ staining, cells were restimulated with phorbol 12-myristate 13-acetate and 400 ng/mL ionomycin for 4 h in the presence of 3 μg/mL of brefeldin A. Afterwards, the cells were fixed and permeabilized overnight using the fixation/permeabilization buffer kit (eBioscience) and then stained with fluorochrome-conjugated mAbs. For sample acquisition, the FACSVerse flow cytometer (Becton Dickinson, Mountain View, CA, USA) and FlowJo software version 7.8. (TreeStar Inc., Ashland, OR, USA) were used for the analyses. Appropriate fluorescence minus one controls or isotype controls were used to establish gates for cell marker positivity.

### 2.6. Nitric Oxide Assay

Mononuclear cells retrieved from SCs (1 × 10^5^/well) were incubated in a 96-well microtiter plate for 2 h in a humidified 5% *v*/*v* CO_2_ atmosphere at 37 °C. Subsequently, the wells were washed twice with PBS and then incubated overnight at 37 °C in a humidified 5% *v*/*v* CO_2_ atmosphere with or without the addition of 1 µg/mL of LPS. The supernatants were analyzed for nitric oxide (NO) content using a method relying on the Griess reaction [30]. The nitrite concentration in the samples was assessed based on optical densities measured at 405 nm (Multiscan Ascent) and calculated using a sodium nitrite standard ranging from 1 to 80 μM.

### 2.7. Superoxide Anion Radical and Oxidative Damage Products

The presence of superoxide anion radical (O2˙^−^) was investigated in brain tissue of EAE rats, according to the modified technique of Pick et al. [31]. All 96 wells in a microtitration plate were charged with 40 μL of the sample and 40 μL of nitro blue tetrazolium chloride. Subsequently, 40 μL NADH and phenazine methosulfate was added to each well. Absorption of reduced tetranitro blue tetrazolium chloride was measured at 532 nm. The results were expressed as nmol/mg protein.

Malondialdehyde (MDA), an indicator of the level of lipid peroxidation, was determined by the following method described by Uchiyama et al. [32]. The method is based on the formation of a colored product after the stoichiometric reaction between thiobarbituric acid (TBA) and various lipid-derived aldehydes. The reaction was carried out by mixing 50 μL sample and 250 μL TBA. The reaction mixture was incubated at 90 °C for 1 h and then cooled to 4 °C in ice. MDA, produced by the hydrolysis of lipid hydroperoxides heated under acid conditions, reacts with TBA to form a complex that absorbs at 532 nm. Results were expressed in mmol/mg tissue.

Oxidative damage of proteins was determined by measuring advanced oxidation protein products (AOPP) following the method of Selmeci et al. [33] with minor modifications. Briefly, 20 μL of the sample was mixed with 180 μL citric acid and 10 μL potassium iodide in a 96-well microplate. The measurements were performed at 532 nm. The results were expressed in mmol/mg tissue.

### 2.8. qRT-PCR

Brain tissue samples were snap-frozen and homogenized in TRIzol extraction reagent (Invitrogen Life Technologies, Carlsbad, CA, USA), and were preserved at −70 °C until RNA purification. Total RNA extraction was conducted using TRIzol reagent following the manufacturer’s guidelines. RNA concentration and purity were assessed via spectrophotometric analysis (OrionTM AquaMate 8000 from Thermo Scientific, Waltham, MA, USA). For cDNA synthesis, the High Capacity cDNA Reverse Transcription Kit (Applied Biosystems, Waltham, MA, USA) was utilized according to the manufacturer’s recommendations. The qRT-PCR reaction mixtures comprised 5 μL of cDNA template, 1× TaqMan Gene Expression Master Mix with Uracil-DNA glycosylase (UDG) (Applied Biosystems), and 1× mix of premade primer and hydrolysis probe sets (TaqMan Gene Expression Assays, Applied Biosystems) in a total volume of 25 μL. Reactions were performed in triplicate under the standard conditions of the Applied Biosystems 7500 Real-Time PCR System. The following TaqMan Gene Expression Assays were employed: *Nrf2* (*Nrf2*, also known as *Nfe2l2*; Rn00582415_m1), *HO-1* (*Hmox1*; Rn00561387_m1), *SOD1* (*Sod1*; Rn00566938_m1), *SOD2* (*Sod2*; Rn00690588_m1), and *β-actin* (*Actb*; Rn00667869_m1). Target mRNA expression was assessed using the SDS v1.4.0. software (Applied Biosystems) and comparative threshold cycle (dCt) method, with β-actin as a reference gene.

### 2.9. Statistical Analysis

Data were presented as mean ± SEM and analyzed using GraphPad Prism 5 software. Two-way ANOVA (treatment × age) followed by a Bonferroni post hoc test was performed for statistical analysis. Statistical significance was set at *p* < 0.05.

## 3. Results

### 3.1. Memantine Ameliorated Clinical Disease with Greater Effect in Aged EAE Rats

**Figure 1:**
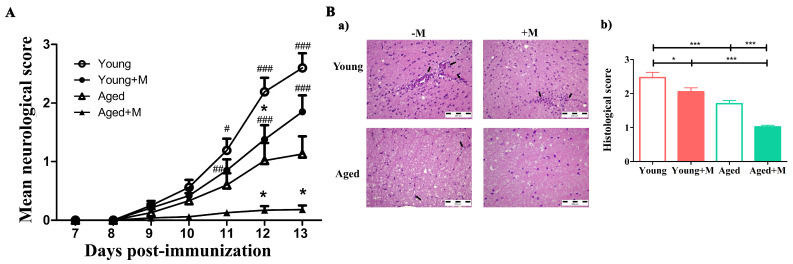
Memantine treatment reduced the mean neurological score and histological score in rats of both age groups. (Panel (**A**)) Line graph indicates the mean daily neurological score of EAE in rats treated with memantine (+M) or administered with vehicle (−M) for 7 consecutive days from day 7 to day 13 post-immunization. Data are presented as mean ± SEM (data from 4 experiments, n = 6 rats/group/experiment), with statistical differences determined using a two-way ANOVA; # *p* < 0.05; ## *p* < 0.01; ### *p* < 0.001; # young vs. aged; * *p* < 0.05; * +M vs. age-matched −M. (Panel (**B**)) Representative photomicrographs (**a**) indicate mononuclear cell infiltrations in spinal cord sections of memantine-treated (+M) or administered with vehicle (−M) young and aged rats. The arrows indicate mononuclear cell infiltrates. Bar graph (**b**) indicates histological scores for spinal cord of memantine-treated and untreated young and aged EAE rats. Histological scoring is described in the Material and Methods section. Data are presented as mean ± SEM (n = 3 rats/group). * *p* < 0.05; *** *p* < 0.001.

In accordance with previous studies [26,27], aged *DA* rats exhibited a substantially lower incidence and mean daily neurological score of EAE compared with young rats (Figure 1). Administration of memantine from 7th day post-immunization (dpi), i.e., before the onset of clinically manifest disease, until the peak of disease (13th dpi) resulted in a lower incidence of clinically manifest disease in young and aged EAE rats (Figure 1). In addition, the mean daily neurological score decreased in memantine-treated EAE rats of both age groups, but this was significant (*p* < 0.05) for aged rats at the 12th and 13th dpi and for young rats only at the 12th dpi (Figure 1). Following the administration of memantine, a reduction in both the maximum neurological score (calculated as the sum of the highest clinical scores divided by the number of rats with clinical EAE) and the cumulative neurological score (calculated as the sum of the daily clinical scores divided by the number of rats with clinical EAE) was observed in young and aged rats, but this reduction was only statistically significant in aged EAE rats (*p* < 0.01 and *p* < 0.05, respectively) (Table 1). In contrast to the young rats, body weight loss was significantly lower (*p* < 0.01) in the treated aged rats compared with the untreated aged rats on the 13th dpi (Table 1). Histopathological analysis showed a significantly lower (*p* < 0.001) histological score in the memantine-treated aged rats in comparison with untreated aged rats (Figure 1). Consistent with this, the mononuclear cell infiltrate retrieved from SCs of memantine-treated aged rats was markedly low (0.45 ± 0.1 × 10^5^) compared with the infiltrate retrieved from untreated aged rats (7.3 ± 0.5 × 10^5^), young untreated rats (27.3 ± 0.9 × 10^5^), or treated rats (22.57 ± 0.6 × 10^5^).

### 3.2. Memantine Reduced the Frequency and Number of CD4^+^ T Lymphocytes Infiltrating SC

**Figure 2:**
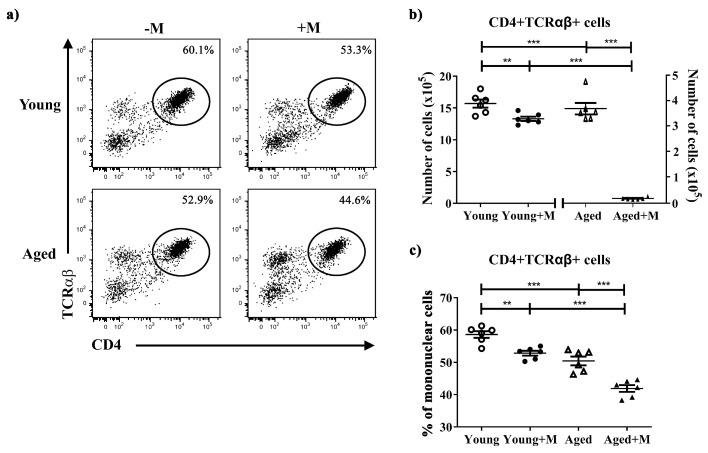
Memantine reduced the frequency and number of CD4^+^ T lymphocytes infiltrating the spinal cord of immunized rats of both age groups. (**a**) Representative flow cytometry dot plots present CD4 vs. TCRαβ staining of mononuclear cells (MNCs) retrieved at the peak of EAE from spinal cords (SCs) of young and aged rats treated with memantine (+M) or vehicle (−M). Numbers in dot plots represent frequency. Scatter plots present (**b**) the number of CD4^+^ TCRαβ^+^ cells and (**c**) their frequency among MNC cells retrieved from SCs. Data are presented as mean ± SEM from 6 rats per group, with statistical differences determined using a two-way ANOVA. ** *p* < 0.01; *** *p* < 0.001.

Irrespective of age, semiprophylactic administration of the NMDAR antagonist significantly reduced the number of CD4^+^ T lymphocytes, the key cells that drive the pathogenesis of EAE. The memantine-induced reduction in the number of CD4^+^ T lymphocytes harvested from the SC was more pronounced in aged (*p* < 0.001) than in young (*p* < 0.01) EAE rats (Figure 2b). In addition, the frequency of CD4^+^ T lymphocytes among SC mononuclear cells was lower in memantine-treated rats in comparison with untreated EAE rats of both age groups, but to a greater extent in aged (*p* < 0.001) than in young rats (*p* < 0.01) (Figure 2a,c).

### 3.3. Memantine Was More Efficient in Reducing Reactivation of CD4^+^ T Lymphocytes Infiltrating the SC in Aged than Young Rats Immunized for EAE

**Figure 3:**
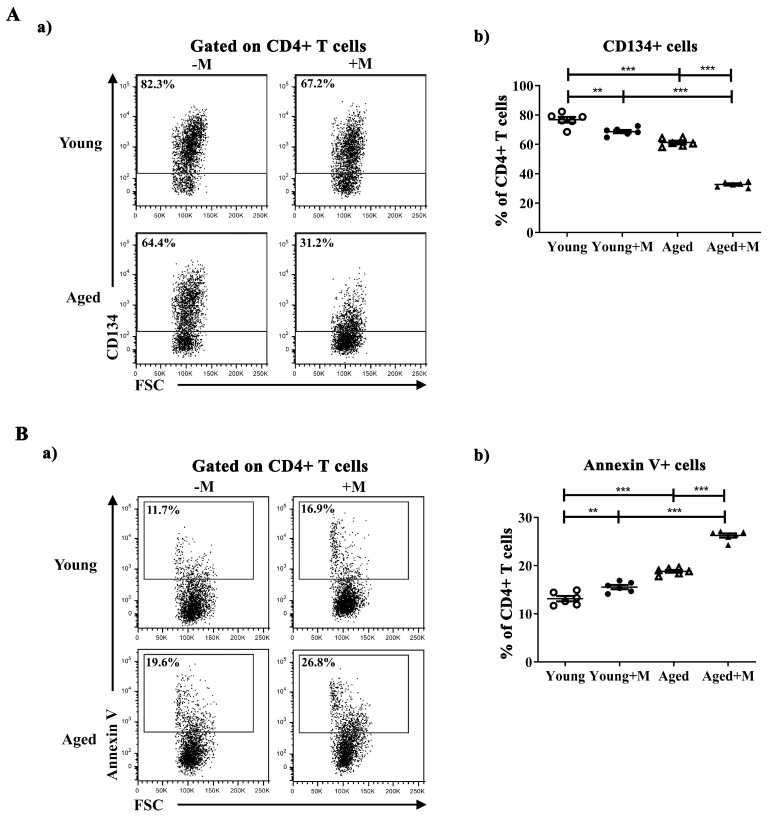
Memantine reduced reactivation and increased apoptosis of CD4^+^ T lymphocytes in the SC more effectively in aged than in young EAE rats. Representative flow cytometry dot plots show (**a**) CD134 (Panel (**A**)) and (**a**) Annexin V (Panel (**B**)) staining of cells gated as CD4^+^ T lymphocytes among mononuclear cells (MNCs) retrieved at the peak of EAE from spinal cords (SCs) of young and aged rats treated with memantine (+M) or vehicle (−M). Numbers in dot plots represent frequency. Scatter plots present the frequency of (**b**) CD134^+^ (Panel (**A**)) and (**b**) Annexin V^+^ (Panel (**B**)) cells among CD4^+^ T cells in SCs from young and aged +M and −M EAE rats. Data are presented as mean ± SEM from 6 rats per group. Two-way ANOVA showed significant interaction between the effects of treatment and age for the frequency of CD134^+^ cells (F_(1,20)_ = 63.22; *p* < 0.001) and Annexin V^+^ cells (F_(1,20)_ = 34.34; *p* < 0.001). ** *p* < 0.01; *** *p* < 0.001.

Infiltrating CD4^+^ T lymphocytes express the costimulatory molecule CD134 in both murine EAE and CNS tissue of human MS patients, which is crucial for their activation and accumulation in the CNS [34]. A pivotal role for the reactivation and expansion of T cells within the CNS is attributed to CD134 signaling, which enables the survival of these cells in the tissue [34]. Memantine reduced the proportion of reactivated CD134^+^ cells among the CD4^+^ T lymphocytes more effectively in SCs from aged (*p* < 0.001) than from young (*p* < 0.01) EAE rats (Figure 3A). Consistent with previous results was a greater increase in the proportion of apoptotic cells among CD4^+^ T lymphocytes in SCs from aged EAE rats (*p* < 0.001) compared with young ones (*p* < 0.01) following memantine administration (Figure 3B).

### 3.4. Memantine Reduced the Proportion of CD4^+^ T Lymphocytes Producing IL-17 or IFN-γ in Aged Rats

**Figure 4:**
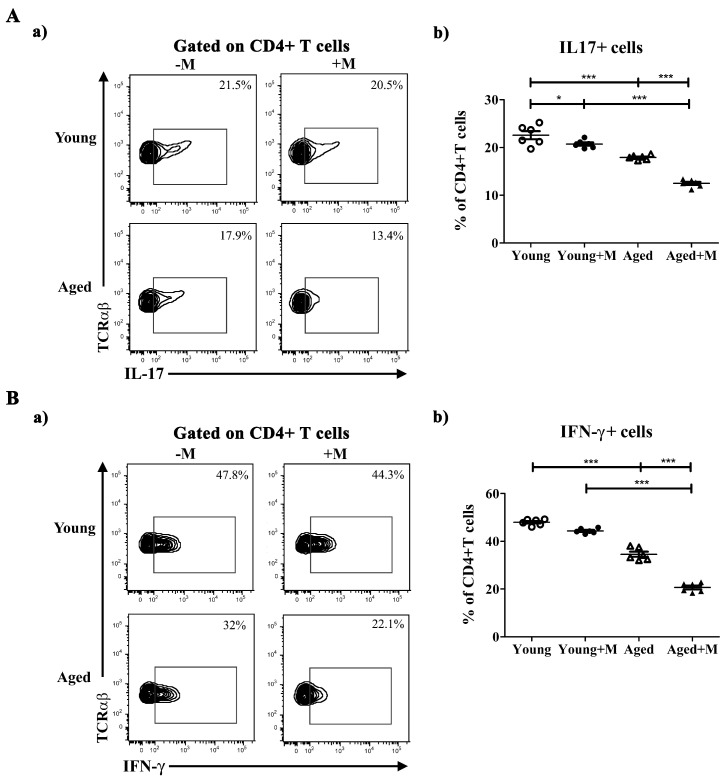
NMDAR antagonist induced lower proportions of IL-17^+^ and IFN-γ^+^ cells among CD4^+^ T cells in SCs of aged rats. Representative flow cytometry dot plots show (**a**) IL-17 (Panel (**A**)) and (**a**) IFN-γ (Panel (**B**)) staining of cells gated as CD4^+^ T lymphocytes among mononuclear cells (MNCs) retrieved from spinal cords (SCs) of young and aged rats treated with memantine (+M) or vehicle (−M) at the peak of EAE. Numbers in dot plots represent frequency. Scatter plots present the frequency of (**b**) IL-17^+^ (Panel (**A**)) and (**b**) IFN-γ^+^ (Panel (**B**)) cells among CD4^+^ T cells in SCs from young and aged +M and −M EAE rats. Data are presented as mean ± SEM from 6 rats per group. Two-way ANOVA showed significant interaction between the effects of treatment and age for the frequency of IL-17^+^ cells (F_(1,20)_ = 12.88; *p* < 0.01) and IFN-γ^+^ cells (F_(1,20)_ = 20.45; *p* < 0.001). * *p* < 0.05; *** *p* < 0.001.

The CD4^+^ T lymphocytes were examined for their IL-17 and IFN-γ production. The proportion of IL-17^+^ cells among CD4^+^ T lymphocytes was lower (*p* < 0.01) in young rats treated with memantine then in age-matched untreated counterparts, while there was no difference in the proportion of IFN-γ^+^ cells among these cells (Figure 4). On the other hand, the proportion of both IL-17^+^ and IFN-γ^+^ cells was significantly lower (*p* < 0.001) in aged rats administered with memantine than in age-matched untreated counterparts (Figure 4).

### 3.5. Memantine Increased the Expression of CX3CR1 on Microglial Cells in SC from EAE Rats

**Figure 5:**
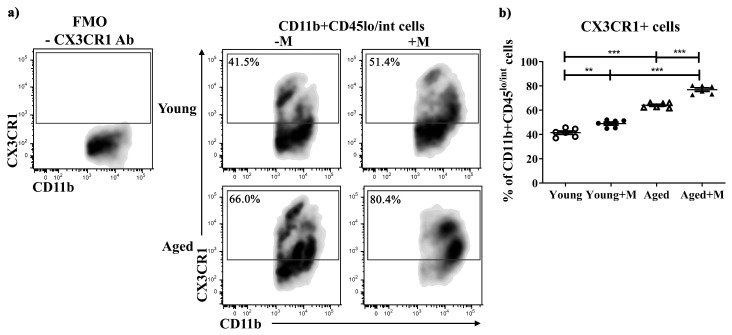
Memantine increased the proportion of CX3CR1-expressing microglia more effectively in aged than in young EAE rats. Representative flow cytometry density plot panels present (**a**) the frequency of CX3CR1^+^ cells among CD11b^+^CD45^lo/int^ microglia retrieved from spinal cord (SC) mononuclear cells (MNCs) at the peak of EAE from young and aged rats treated with memantine (+M) or vehicle (−M). Numbers in dot plots represent frequency. FMO control incubated with secondary antibody (Ab) alone, without anti-CX3CR1 Ab (-CX3CR1 Ab), was used to settle the gating boundary for CX3CR1^+^ cells. (**b**) Scatter plots indicate the frequency of CX3CR1^+^ cells among CD11b^+^CD45^lo/int^ microglia. Data are presented as mean ± SEM from 6 rats per group. Two-way ANOVA showed significant interaction between the effects of treatment and age for CX3CR1^+^ microglial cells (F_(1,20)_ = 4.58; *p* < 0.05). ** *p* < 0.01; *** *p* < 0.001.

The fractalkine receptor (CX3CR1) is present exclusively on microglial cells in the CNS [34,35]. Disruption of CXCL1/CX3CR1 signaling is considered to be one of the most important elements in the pathogenesis of CNS-related disorders [36,37]. Pro-inflammatory microglia activation results in a loss of CX3CR1 [38]. Furthermore, the phagocytic capacity of microglia is significantly reduced in CX3CR1 gene knockout mice, resulting in the persistence of myelin debris and impaired remyelination [39]. Based on the surface expression of the CD45 molecule, we distinguished pro-inflammatory monocytes that infiltrated the SC (CD11b^+^CD45^high^) and microglia (CD11b^+^CD45^low/int^). We found that memantine-induced increase in the frequency of CX3CR1^+^ cells among CD11b^+^CD45^lo/int^ cells was less pronounced in SCs from young (*p* < 0.01) than aged (*p* < 0.001) EAE rats (Figure 5).

### 3.6. NMDAR Antagonist Reduced Nitric Oxide Production by Macrophages/Microglia from the SC of Aged EAE Rats

To investigate the impact of the NMDAR antagonist on the NF-κB/iNOS/NO signaling pathway in macrophages/microglia from EAE rats, we stimulated mononuclear cells retrieved from SCs of EAE rats with LPS and examined their NO production. NF-κB is the major transcription factor for the expression of genes involved in inflammatory responses induced by LPS activation. In fact, NO increases its production as a result of gene expression of iNOS, which is mainly regulated by NF-kB [40].

**Figure 6:**
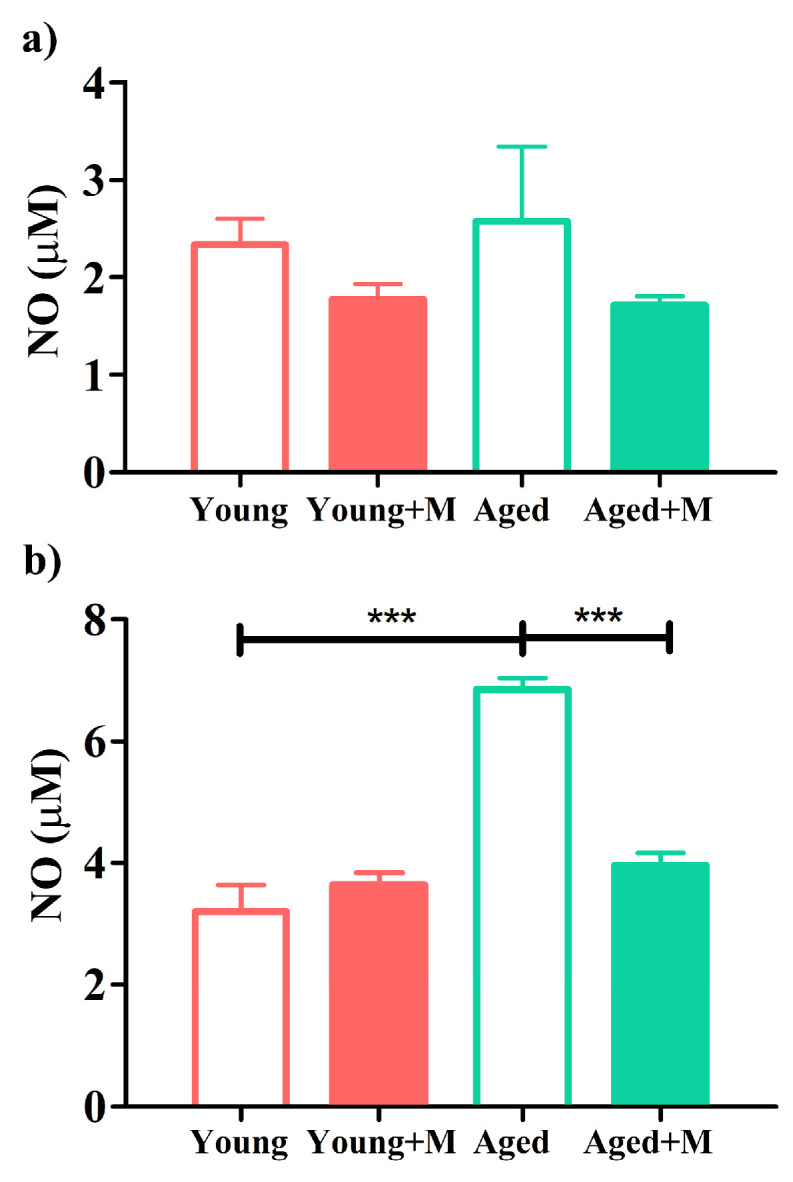
Memantine reduced the production of nitric oxide by macrophages/microglia obtained from the spinal cord of aged EAE rats. Bar graphs indicate concentration of nitric oxide in overnight mononuclear spinal cord (SC) cell cultures (**a**) in the medium alone or (**b**) stimulated with lipopolysaccharide (LPS). Data are presented as mean ± SEM from 6 rats per group. Two-way ANOVA showed significant interaction between the effects of treatment and age for NO concentration determined in LPS-stimulated SC mononuclear cell cultures (F_(1,20)_ = 30.50; *p* < 0.001). *** *p* < 0.001.

In the absence of LPS, there was no difference in NO production from SC mononuclear cells retrieved from untreated and treated rats of both age groups (Figure 6a). On the other hand, NO production was higher (*p* < 0.001) in LPS-stimulated SC mononuclear cells from aged than from young untreated rats. Moreover, NO production was significantly decreased (*p* < 0.001) by LPS-stimulated mononuclear cells harvested from SCs of memantine-treated aged rats, whereas this effect was absent in LPS-stimulated mononuclear SC cells from young memantine-treated rats (Figure 6b).

### 3.7. Memantine Was More Efficient in Reducing Oxidative Brain Tissue Damage in Aged Rats

**Figure 7:**
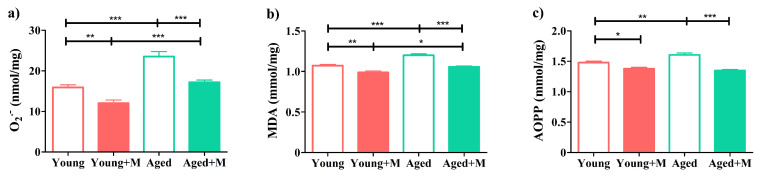
Memantine was more efficient in reducing oxidative brain tissue damage in aged rats. Bar graphs indicate levels of (**a**) O2˙^−^, (**b**) malondialdehyde (MDA), and (**c**) advanced oxidation protein products (AOPPs) at the peak of EAE in the brain tissue of young and aged EAE rats treated with memantine (+M) or vehicle (−M). Data are presented as mean ± SEM from 6 rats per group. Two-way ANOVA showed significant interaction between the effects of treatment and age for the level of AOPP (F_(1,20)_ = 12.19; *p* < 0.01). * *p* < 0.05; ** *p* < 0.01; *** *p* < 0.001.

There are numerous findings that point to a significant role of oxidative stress in the pathogenesis of MS and its animal model EAE [41,42]. On the other hand, reactive oxygen species (ROS) play an important role in the aging process. During the aging process, increased ROS production is accompanied by a reduction in antioxidant defense [43,44,45]. Here, the level of superoxide anion radicals (O2˙^−^) was higher (*p* < 0.001) in the brain tissue of aged rats than in the brain tissue of young rats. However, the NMDAR antagonist reduced O2˙^−^ levels to a greater extent in aged rats (*p* < 0.001) than in young rats (*p* < 0.01) (Figure 7). In addition, brain tissue from EAE rats was analyzed for the levels of MDA, a marker of lipid peroxidation, and AOPP, a reliable marker of the degree of oxidant-induced protein damage. Higher levels of MDA and AOPP were found in the brain tissue of aged rats compared with their levels in brain tissue of young EAE rats (*p* < 0.001; *p* < 0.01; respectively). Memantine treatment led to a reduction in MDA and AOPP levels in the brain tissue of treated EAE rats of both ages, but again to a greater extent in aged (*p* < 0.001) than in young rats (*p* < 0.01; *p* < 0.05; respectively) (Figure 7).

### 3.8. Antagonizing NMDARs Induces an Age-Dependent Modulation of Brain Antioxidant Defense in EAE Rats

**Figure 8:**
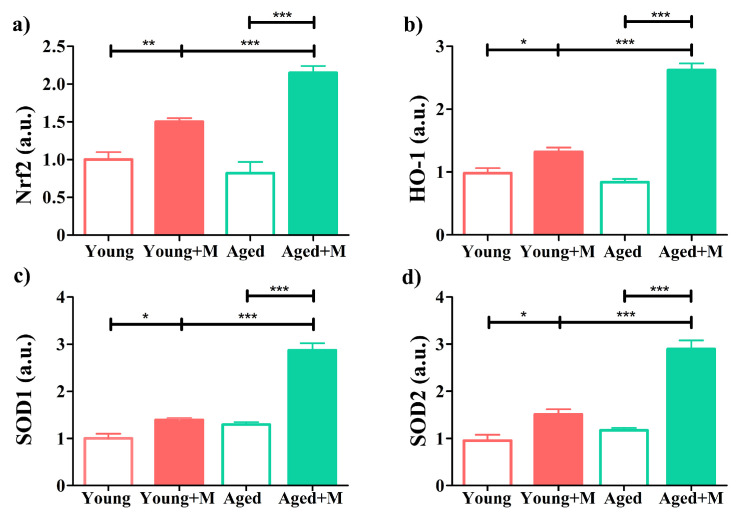
Memantine increased the expression of mRNAs for Nrf2 and Nrf2-regulated enzymes. Bar graphs show the fold change in (**a**) nuclear factor (erythroid-derived 2)-like 2 (Nrf2), (**b**) heme oxygenase 1 (HO-1), (**c**) superoxide dismutase (SOD)1, and (**d**) SOD2 mRNA expression in brain tissue at the peak of EAE from young and aged rats treated with memantine (+M) or vehicle (−M), as determined by qRT-PCR. Results are normalized to β-actin expression. Data are presented as mean ± SEM from 6 rats per group. Two-way ANOVA showed significant interaction between the effects of treatment and age for Nrf2 (F_(1,20)_ = 127.0; *p* < 0.001), HO-1 (F_(1,20)_ = 80.06; *p* < 0.001), SOD1 (F_(1,20)_ = 37.91; *p* < 0.001), and SOD2 (F_(1,20)_ = 22.45; *p* < 0.001) mRNA expression. * *p* < 0.05; ** *p* < 0.01; *** *p* < 0.001.

Nrf2 is considered to be the major transcription factor that regulates antioxidant response element-mediated gene expression in order to regulate the physiologic and pathophysiologic outcomes of oxidant exposure [46,47]. Considering that (i) low Nrf2 levels in the CNS are the most likely cause of the inability to elicit a strong antioxidant response in EAE [48]; (ii) memantine promotes the expression of the NRF2/HO-1 antioxidant signaling pathway [49]; and (iii) memantine upregulates *SOD1* and *SOD2*, antioxidant genes regulated by Nrf2 transcription factor [47,50], in brain tissue of EAE rats [24], the expression of Nrf2, HO-1, SOD1, and SOD2 mRNAs in brain tissue was examined. There was no significant difference in the expression of mRNA for Nrf2 in the brain tissue of untreated young compared with aged EAE rats (Figure 8). Memantine increased the expression of mRNA for Nrf2 in EAE rats of both age groups, but this was more pronounced in aged (*p* < 0.001) than in young rats (*p* < 0.01) (Figure 8). In agreement with this finding, the expression of HO-1, SOD1, and SOD2 mRNAs was increased in the brain tissue of young (*p* < 0.05) and aged (*p* < 0.001) EAE rats after memantine administration, although this was more pronounced in aged EAE rats (Figure 8).

## 4. Discussion

The role of NMDARs expressed on neurons in the pathogenesis of MS and its animal model EAE has been recognized [51,52]. However, data on the influence of aging on the role of these receptors in MS are scarce [53]. To the best of our knowledge, the impact of aging on the contribution of NMDARs expressed on immune cells in MS has not yet been investigated. Our results showed an age-dependent involvement of NMDARs in the pathogenesis of EAE, which was more pronounced in aged rats than in young ones. The effects of antagonizing NMDARs with memantine, a non-competitive antagonist, were reflected in a lower incidence and a significantly milder disease course, according to maximum and cumulative neurological scores, in aged EAE rats compared with untreated age-matched EAE rats. On the other hand, these neurological scores in young animals were slightly decreased after memantine administration, but not statistically significant. Memantine decreased the number of CD4^+^ T lymphocytes, the major effector cells in EAE [54], retrieved from the SC of immunized rats. This could be due to restricted permeability of the blood–brain barrier, and reduced reactivation of encephalitogenic CD4^+^ T cells in the CNS and their increased apoptosis. It has already been shown that semiprophylactic administration of memantine significantly restores blood–brain integrity [29]. In this study, we showed for the first time that memantine reduced the expression of the tumor necrosis factor receptor CD134 (OX40) on CD4^+^ T lymphocytes in EAE rats. Furthermore, this effect was more prominent in aged rats. The signaling through the CD134 molecule plays a crucial role in the establishment of autoimmune encephalitis [34,55]. In myelin-immunized rats, CD134 denoted those T cells that are specific for myelin. When T cells arrive in the CNS, surface expression of the CD134 molecule is upregulated, presumably as a consequence of antigen recognition in the target organ [56]. Ligation of CD134 expands the antigen-specific T cell pool, leads to increased production of effector cytokines, and prolongs the activation and survival of T cells in the effector phase of EAE [34,55,57]. In addition, T cells activated through CD134 effectively counteract the suppression mediated by regulatory T cells [58]. However, cells expressing CD134 have been found in the inflammatory CNS infiltrates of MS patients [34]. Recent data corroborate the involvement of CD134-CD134L signaling in the pathogenesis of MS [59]. Consistent with the finding that the NMDAR antagonist memantine reduces CD134 expression on CD4^+^ T lymphocytes, increased apoptosis of these cells was observed. NMDAR antagonists, including memantine, have been shown to have profound effects on T cell function [60]. Activation of human, as well as murine, CD4^+^ T lymphocytes leads to an upregulation of functional NMDARs on their surface [20,21]. Furthermore, it has been demonstrated that all T lymphocytes infiltrating the region of neuroinflammation, induced by SC injury, express NMDARs [21]. Triggering of NMDARs expressed on CD4^+^ T lymphocytes by relevant ligands has differential effects on cytokine production, proliferation, and survival of these cells [20]. Our results showed a lower frequency of IL-17^+^ and IFN-γ^+^ cells among CD4^+^ T lymphocytes in the SC of aged rats treated with memantine compared with untreated aged rats. The pathological activity of NMDARs is abrogated by memantine, but it is also possible that the immunosuppressive effect of the NMDAR antagonist is mediated by a reduction in the conductance of the K_V_1.3 and K_Ca_3.1 potassium channels in activated CD4^+^ T cells [60].

The expression of NMDARs in nascent and activated microglia in the human and murine CNS has been demonstrated [61,62]. Actually, NMDAR stimulation is a mechanism for microglial activation [18,61,63]. In a vicious cycle, damaged neurons can activate microglial NMDARs. Stimulation of microglial NMDARs leads to pro-inflammatory polarization and the expression of pro-inflammatory cytokines as well as the release of factors such as reactive oxygen species (ROS) and NO [18,61,62]. Thus, microglia contribute to neuroinflammation and neurodegeneration. Autoimmunity against myelin components leads to the activation of microglia, which can release glutamate, pro-inflammatory cytokines, ROS, and NO. In this way, the death of oligodendrocytes can be induced and myelin destroyed [64]. Direct stimulation of microglial NMDARs with NMDA led to a 15-fold increase in the expression of iNOS, a classic marker of the pro-inflammatory microglial phenotype, with a corresponding six-fold increase in NO production [18]. Antagonists of NMDARs suppress the production of iNOS, NO, and pro-inflammatory cytokines by microglia as well as the nuclear translocation of NF-κB in hypoxic microglia/oligodendrocyte co-cultures, thus maintaining oligodendrocyte survival [62]. The microglia-expressed fractalkine receptor CX3CR1 is highly abundant in the healthy CNS and is a key regulator of microglial neurotoxicity. During EAE, CX3CR1 deficiency leads to increased microglia inflammatory behavior, disease exacerbation with severe inflammation, and neuronal loss [35,65]. In the present study, we found an increased percentage of CX3CR1-expressing microglia in the SC after memantine administration, but to a greater extent in aged EAE rats. In accordance with this, there was a significant reduction in NO production by macrophages/microglia harvested from the SC of memantine-treated aged rats compared with NO production by macrophages/microglia in untreated aged rats. Since this was not the case in young animals, it could be concluded that NO synthesis by microglia plays a greater role in the pathogenesis of EAE in aged than in young animals. This is also supported by the higher NO production by macrophages/microglia in untreated aged rats compared with young ones.

Brain tissue is very sensitive to the action of ROS, due, among other reasons, to its high demand for oxygen, high lipid content, modest endogenous antioxidant defense, and neurotransmitter autoxidation [66]. Both microglia and macrophages produce ROS during MS [66]. There is ample evidence that increased production of ROS is an important cause of tissue damage during MS and EAE [41,67,68,69]. Activation of the Nrf2–ARE pathway may play a protective role in the pathogenesis of these disorders by inducing antioxidant enzymes such as HO-1, SOD1, SOD2, and others that might prevent oxidative damage to neurons and oligodendrocytes [70,71]. We were able to show that memantine reduces the formation of superoxide anion radicals and the level of MDA, an end product of lipid peroxidation, as well as the intensity of oxidative protein modification, measured by AOPP, in the brain tissue of aged EAE rats more efficiently than in young EAE rats. Consistent with this finding, increased expression of mRNAs for Nrf2 and Nrf2-regulated enzymes (HO-1, SOD1, and SOD2) was found in memantine-treated EAE rats compared with untreated EAE rats, which was again more pronounced in aged rats. The enzyme SOD2, the mitochondrial isoform, is of particular importance for neurodegenerative diseases [72]. In fact, it has been demonstrated that neurons are unable to survive a genetic deletion of SOD2 [66]. It should also be mentioned that there was no significant difference in the expression of Nrf2 and Nrf2-regulated enzymes between young and aged untreated EAE rats, which did not correspond with the parameters of oxidative damage. Actually, MDA and AOPP levels were higher in brain tissue of aged than young EAE rats. This result could be ascribed to an aging-induced increase in these biological markers of oxidative stress [73,74].

To date, there is insufficient evidence for the efficacy of memantine, a non-competitive NMDAR antagonist, in MS patients in terms of preventing cognitive deterioration, controlling spasticity, reducing fatigue, and improving the degree of functionality compared with placebo [75]. However, elderly patients were underrepresented in clinical trials investigating the potential benefits of memantine in MS patients [75]. Emerging evidence suggests that the survival rate of MS patients has increased, as has the proportion of patients with late and very-late onset [4,5,6,7,8]. Chronological age has been associated with the rate of non-relapse-related disability accumulation in MS [9]. Therefore, it is extremely important to understand the impact of aging on the pathophysiologic mechanisms responsible for the development and progression of MS. This study provides insight into the impact of aging on the role of NMDARs in an animal model of MS, suggesting that targeting NMDARs in the elderly might be more beneficial than in younger patients with MS.

## 5. Conclusions

Collectively, these findings indicate that NMDARs play a more important role in the pathogenesis of EAE in aged than in young rats. Although further research is undoubtedly needed, particularly on the role of NMDARs on immune cells, these results suggest that pharmacological inhibition of NMDARs might be of greater importance in elderly MS patients.

## Figures and Tables

**Table 1:** Effects of memantine on EAE parameters.

	Incidence (%)	Maximal Neurological Score	Cumulative Neurological Score	Body Weight Loss (%) on 13th dpi
Young	100	2.6 ± 0.25	6.7 ± 0.85	20.7 ± 1.2
Young + M	87.5	2.1 ± 0.27	5.4 ± 0.88	18.6 ± 0.83
Aged	41.7	2.7 ± 0.3	8.1 ± 1.34	6.3 ± 0.77 ^###^
Aged + M	25	0.75 ± 0.11 **^;^ ^#^	2.25 ± 0.6 *	2.1 ± 0.51 **^;^ ^###^

Mean values ± SEM from 24 rats per group are presented. ^#^ *p* < 0.05; ^###^ *p* < 0.001; ^#^ young vs. aged; * *p* < 0.05; ** *p* < 0.01; * +M vs. age-matched −M.

## Data Availability

Dataset available on request from the authors.
